# Automatic generation of creative text in Portuguese: an overview

**DOI:** 10.1007/s10579-023-09646-3

**Published:** 2023-05-03

**Authors:** Hugo Gonçalo Oliveira

**Affiliations:** CISUC, Department of Informatics Engineering, University of Coimbra, Coimbra, Portugal

**Keywords:** Computational creativity, Poetry, Humour, Riddles, Headlines, Language resources

## Abstract

Creativity is an inherently human skill, and thus one of the goals of Artificial Intelligence. Specifically, linguistic computational creativity deals with the autonomous generation of linguistically-creative artefacts. Here, we present four types of text that can be tackled in this scope—poetry, humour, riddles, and headlines—and overview computational systems developed for their generation in Portuguese. Adopted approaches are described and illustrated with generated examples, and the key role of underlying computational linguistic resources is highlighted. The future of such systems is further discussed together with the exploration of neural approaches for text generation. While overviewing such systems, we hope to disseminate the area among the community of the computational processing of the Portuguese language.

## Introduction

Computational creativity (CC) is a branch of Artificial Intelligence (AI) that “studies and exploits the potential of computers to act as autonomous creators in their own right” (Veale & Cardoso, 2019). Despite the lack of a highly consensual definition, one of its main goals is the development of computational systems that exhibit behaviours that would be deemed as creative by unbiased observers (Colton & Wiggins, 2012), often rendered in the form of novel artefacts in fields like the visual arts, music, computer games, literature or linguistics in general. In the latter, produced artefacts are in the form of human language, which is why, to some extent, they can also be seen as a specific application of Natural Language Generation (Gatt & Krahmer, 2018). Artefacts covered include stories, poems, jokes or slogans, among others, which can be written in different languages. Though not always in the domain of CC, the generation of such artefacts has been receiving more and more attention in recent years, with the increasing popularity of AI and the broad adoption of deep neural networks for text generation. Beyond its applications to entertainment, this kind of research is further motivated by the role of creativity for definiting intelligence, and on the will to test the boundaries of AI.

In this paper, we overview computational systems that generate creative text in the Portuguese language. When compared to other languages, the amount of such systems is scarce. Specifically, for English, several new linguistically-creative systems are developed every year, which results in the existence of countless poetry generators, as well as many systems that produce other types of creative text (see Sects. 2.1, 3.1, 4.1, 5.1 for references). In a minority of cases, this might be due to the lack of resources for Portuguese. Even if the situation is not exclusive of Portuguese, we believe that it is mostly a consequence of a much smaller community and, possibly, less awareness of those that work with the language. This is something that we want to start inverting with this paper. We thus overview systems of this kind, developed in the last 16 years for producing four different kinds of creative artefacts in Portuguese, namely: song lyrics and poetry; Internet humor (i.e., so-called memes); riddles; and (creative) headlines.

Besides being textual artefacts, when produced by humans, all of the previous require creativity. Yet, they are different in aspects like form, exploited knowledge or initial stimulus, and thus their generation ends up following different requirements. We review the specificities of each and describe approaches adopted for their generation, with a focus on the linguistic resources and tools exploited for processing stimuli and selecting the contents to use in their output. For instance, poetry has rhythmic constraints and may take advantage of rhymes; in Internet memes, there must be a connection between a short text and an image; riddles typically have a question-answer format; and headlines are often short texts. At the same time, the generation process may be guided by an initial set of words, a sentiment or recent news, which may be reflected in the resulting text. For achieving this purpose, creative systems exploit resources like corpora, morphology or sentiment lexicons, semantic networks or word embeddings, as well as tools for syllable division, part-of-speech (PoS) tagging or lemmatization. Examples of produced results are also presented and analysed.

The aim of this paper is thus twofold:

- We introduce work on linguistic CC, an emerging area, not always seen as a serious research effort, hopefully raising awareness to those potentially interested in exploring some of the described tasks, especially if they are willing to do it in Portuguese;
- We describe (unconventional) applications of available computational linguistic resources and tools for Portuguese, specifically in the domain of linguistic CC.

Following this introduction, this paper has four sections, each targeting a different kind of creative artefact. Sections start with a brief reference to some related work. This is followed by a description of one or more systems and their approaches for generating the target artefacts in Portuguese, namely: Sect. 2 is on the generation of poetry and song lyrics. Since more than one system was developed for this, the section refers to some older and some more recent systems, in what can be seen as a chronological perspective; Sect. 3 is on the generation of Internet memes; Sect. 4 is on the generation of creative riddles; Sect. 5 is on the generation of creative headlines. After this, Sect. 6 discusses the differences and similarities between different artefacts, associated constraints, as well as the resources and tools used for handling them, also including a brief reference to evaluation. Section 7 bridges between what was overviewed and the current state-of-the-art of text generation, with a discussion on how these systems could evolve and an exploration of recent models; Sect. 8 concludes the paper, while highlighting applications of linguistic CC and setting future research directions.

## Poetry and song lyrics

Poetry expresses feelings and ideas in general, through different forms and shapes. In more traditional forms, poetry can be guided by formal constraints, which is reflected in how lines are grouped and on their metre. Song lyrics can be seen as a kind of poetry that aims to be sung, also making the former more accessible. They are closely attached to the song’s rhythm, but with more varied forms, when compared to traditional poetry. In fact, in the same lyrics, it is more common to have lines of different lengths, grouped in stanzas of different sizes. As for traditional poetry, classic forms are typically followed (e.g., the sonnet or the haiku), each setting specific regularities in the metre, lines per stanza, or presence and position of rhymes.

After an overview of related work, this section briefly presents how the automatic generation of traditional forms poetry and of song lyrics was tackled in Portuguese. The main focus are two systems, Tra-la-Lyrics (Gonçalo Oliveira et al., 2007) and PoeTryMe (Gonçalo Oliveira, 2012), and adaptations of the latter.

### Related work

Poetry generation is a popular task in the CC domain, with a broad range of AI techniques applied, considering different features, forms, and languages (Gonçalo Oliveira, 2017b; Lamb et al., 2017). Such techniques include, but are not limited to, case-based reasoning (Gervás, 2001), evolutionary algorithms (Manurung, 2003), multi-agent systems (Misztal & Indurkhya, 2014), generative summarization (Yan et al., 2013), or finite-state automata (Ghazvininejad et al., 2016). In the last decade, poetry generation adopted recent trends for text generation in general, namely deep neural networks. This includes RNN language models (Van de Cruys, 2020; Zhang & Lapata, 2014), which can generate the next lines from the previous ones; conditional variational auto-encoders (Chen et al., 2019; Yang et al., 2018), where the user intent can be represented by keywords that condition the output; or transformers (Liao et al., 2019; Takeishi et al., 2022; Zhao & Lee, 2021), fine-tuned in data in the target style.

As for languages, besides English (e.g., Colton et al., 2012; Ghazvininejad et al., 2016; Manurung, 2003; Veale, 2013), there are dozens of reported generators of Chinese traditional poetry (e.g., Liao et al., 2019; Yan et al., 2013; Yang et al., 2018; Zhang & Lapata, 2014). Though in a much smaller amount, we highlight work on poetry generation in Latin languages, like Portuguese, namely in Spanish (Gervás, 2001), Italian (Zugarini et al., 2019) and French (Van de Cruys, 2020).

Poetry generation is a complex task because it generally considers different levels of language, from phonetics, to syntax and semantics. Yet, it does not have to be extremely precise (Gervás, 2000), and not all levels need to be addressed equally, because several rules, typical in natural language, can or actually need to be broken (Manurung, 2003). Three main properties should hold in poetic text (Manurung, 2003): grammaticality, meaningfulness, poeticness.

*Grammaticality* can be controlled by a grammar, which might, in some cases, account for semantics (Manurung, 2003). Common alternatives are to use templates of human-written poems (Colton et al., 2012; Toivanen et al., 2014), or to rely on language models (Ghazvininejad et al., 2016; Liao et al., 2019; Zhang & Lapata, 2014), both expected to meet syntactic rules.

Towards *meaningfulness*, an initial input has to be provided. This can be in the form of keywords for defining a topic (Ghazvininejad et al., 2016; Zhang & Lapata, 2014), or a full textual document, such as a news story (Colton et al., 2012; Toivanen et al., 2014), where input keywords or additional information are acquired from. Recently, images have also been used as the input for neural poetry generation (Liu et al., 2018). To handle semantics or expanding the set of keywords, more traditional systems resort to semantic networks (Agirrezabal et al., 2013; Colton et al., 2012), like WordNet (Fellbaum, 1998), or models of distributional semantics (Ghazvininejad et al., 2016; Toivanen et al., 2014).

Still, many human-written poems are open to different interpretations. Therefore, in traditional poetry, a less clear message can sometimes be compensated by the presence of formal features. Form, which is reflected in how lines are grouped, metric regularities, and the presence of rhymes, contributes to *poeticness*. Also relevant is figurative language, which can be implicitly present, due to the reuse of fragments from human-written poetry, but may also be specifically addressed (Liu et al., 2019; Veale, 2013), as well as features like sentiment (Chen et al., 2019; Colton et al., 2012) or emotion (Misztal & Indurkhya, 2014).

Despite the existence of more open forms, and a stronger connection with the rhythm, song lyrics are not so different from poetry. So, most of the previous techniques and features would apply for their generation. Tra-la-Lyrics (Gonçalo Oliveira et al., 2007) was an early attempt at the automatic generation of text based on rhythm, in this case, in Portuguese. It was mostly based on rules (see Sect. 2.2). Yet, nowadays, a popular approach is to learn neural language models from corpora of lyrics aligned with their melodies (Watanabe et al., 2018), or just lyrics, for the case of rap (Nikolov et al., 2020; Potash et al., 2015). Other approaches generate: lyrics in the style of an artist, with constrained Markov processes (Barbieri et al., 2012); parodies based on the replacement of content words in original lyrics with others related to news headlines (Gatti et al., 2017); both music and lyrics at the same time (Toivanen et al., 2013).

### Tra-la-Lyrics

Tra-la-Lyrics (Gonçalo Oliveira et al., 2007) analyses the rhythm of a melody and generates sequences of matching words. Different strategies can be followed, but the key is to pair each syllable of a word with a note of the melody, and to match stressed syllables with strong beats. Melodies are provided in the ABC music notation,^1^ and distribution of strength across beats is done for a set of common metres, according to Lerdahl and Jackendoff (1983).

For each sequence of beats in the melody, Tra-la-Lyrics selects suitable words from a database, one after another, constrained by the rhythm and other features. A set of constraints and considered features make up a strategy. The simplest strategy has only one additional feature: it tries to use words with the same termination (i.e., rhyme) in specific positions. With this strategy, lyrics match the rhythm well, but lack syntactic and semantic coherence.

In order to provide syntactic coherence, another strategy is based on a grammar with accepted sequences of PoS. This way, in addition to the rhythm, words are constrained by a PoS and inflection. A third strategy, generate & test, uses the same grammar but does not select one word at the time. Instead, it generates a set of complete sentences and selects those that better match the rhythm of the musical phrases.

Having in mind the importance of word and sound repetition, a probability can be additionally set for, when possible, reusing words. Moreover, although the generated lyrics tend to have no semantic coherence, in an attempt to generate text on a desired topic, selection can favor words with lemmas in a given list. Yet, since constraints can be too many for finding matching words, in any strategy, they have a priority, and some can be dropped for increasing the chance of matching the rhythm.

When Tra-la-Lyrics was originally developed, words for the lyrics were obtained from the Floresta Sintá(c)tica (Afonso et al., 2002) treebank, and the idea was to rely on the syntactic annotations for deciding where to use the words. However, for higher flexibility, Floresta Sintá(c)tica ended up being used merely as a repository of words, with their possible PoS tags obtained with Jspell (de Almeida & Pinto, 1994), a morphological analyser for Portuguese. The database also included other properties of the words, such as their lemma, inflection, syllable division and termination. The latter were obtained with SilabasPT,^2^ a library developed specifically for the purpose.

**Fig. 1:**
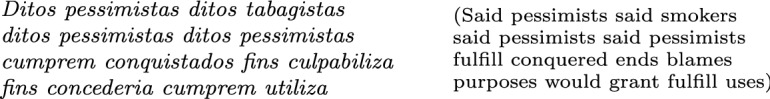
Lyrics produced by the simple Tra-la-Lyrics strategy for the melody of the Portuguese song *Papagaio Louro*

**Fig. 2:**
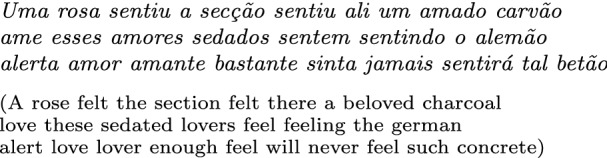
Lyrics produced by the grammar Tra-la-Lyrics strategy for the melody of the Portuguese song *O Barquinho*, with topical words *sentir*, *amor*, *amar* and *rosa*

**Fig. 3:**
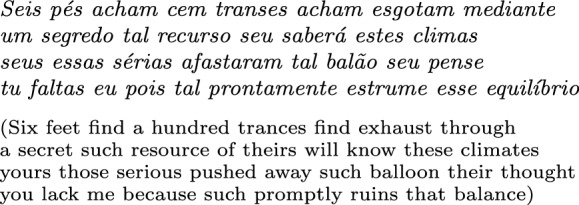
Lyrics produced by the generate and test Tra-la-Lyrics strategy for the melody of the song *Michelle*, by the Beatles

Figures 1, 2, 3 illustrate the outputs of Tra-la-Lyrics for melodies of different original songs, respectively with the simple, the grammar and the generate & test strategy. The first example is to be sung with the melody of the Portuguese song *Papagaio Louro*. Despite not following any syntactic structure, the combination of repetition (words *ditos* and *pessimistas*) and rhymes (*tabagistas* with *pessimistas*, *culpabiliza* with *utiliza*) with a matching rhythm leads to an interesting and somehow funny result.

The second example is for the melody of the Portuguese song *O Barquinho*. This time, rhythm is not matched by one word (*sentindo*), but lyrics are grammatical, which is one step towards coherence. Three words rhyme (*carvão*, *alemão* and *betão*) and several were selected due to the given list of topical words: *sentir*, *amor*, *amar* and *rosa*.

The third example is for the melody of the song *Michelle*, by The Beatles. This time, there are more words not matching the rhythm (e.g., *acham*, *mediante*, *prontamente*) and no rhymes. Moreover, even if the sequence of words follows a grammar, the choice was poor and resulted in odd lines.

### PoeTryMe

Tra-la-Lyrics had a strong focus on the rhythm, and semantic-coherence was not a major concern, also due to the lack of resources at the time. But a few years later, the development of PoeTryMe (Gonçalo Oliveira, 2012) started, with one of the main goals being an improvement of semantic coherence. Instead of following any given rhythm, text produced by PoeTryMe follows structures of poems, characterised by groups of lines (stanzas) and their metre, based on the number of syllables in each. Its key components are a semantic grammar and a semantic network, together with a generation strategy that interacts with them. Specifically, in the semantic network, words are connected according to semantic relations, whereas the semantic grammar is a set of natural language patterns that transmit the relations in the network. Examples of these patterns are in Table 1. They are instantiated for a relation instance and have two gaps, to be filled by words for which the relation holds. The grammar can be edited manually, but most of its patterns were automatically acquired from human-written poetry, with the help of the semantic network. For example, pattern #4 was acquired due to the relation partOf(*versos*, *quadras*). Once in the grammar, it can be instantiated for other instances of the same relation, like partOf(*minutos*, *horas*) or partOf(*degraus*, *escadas*), respectively resulting in the following lines:

- *as minhas horas têm três minutos* (*my hours have three minutes*)
- *as minhas escadas têm três degraus* (*my stairs have three steps*)

Or pattern #7, originally acquired due to the relation propertyOf(*letal*, *morte*), can be instantiated, e.g., for propertyOf(*desastrosa*, *ruína*) or propertyOf(*passional*, *paixão*), resulting in:

- *a ruína é branda e desastrosa* (*the ruin is soft and disastrous*)
- *a paixão é branda e passional* (*the passion is soft and passionate*)

**Table 1:** Patterns in PoeTryMe’s semantic grammar

#	Pattern
1	$\texttt {ADJ}\_\texttt {SYNONYM}\_\texttt {ADJ}\,{::=}<\texttt {arg1}>\texttt {a espada j}\acute{\texttt {a}} <\texttt {arg2}>\texttt {a armadura}$
	// <arg1> the sword already <arg2> the armor
2	$\texttt {ADJ}\_\texttt {ANTONYM}\_\texttt {ADJ}\,{:}{:}{=} \texttt {tudo} \acute{\texttt {e}}<\texttt {arg1}>, \texttt {s}\acute{\texttt {o}} \texttt {eu} <\texttt {arg2}>$
	// everything is <arg1>, only I <arg2>
3	$\texttt {N}\_\texttt {HYPERNYM}\_\texttt {N}\,{:}{:}{=} \texttt {e a pr}\acute{\texttt {o}}{} \texttt {pria}<\texttt {arg2}>\texttt {melhor fora} <\texttt {arg1}>$
	// and the <arg2> itself better was <arg1>
4	$\texttt {N}\_\texttt {PART-OF}\_\texttt {N}\,{:}{:}{=} \texttt {as minhas}<\texttt {arg2}>\texttt {t}\hat{\texttt {e}}{} \texttt {m tr}\hat{\texttt {e}}{} \texttt {s} <\texttt {arg1}>$
	// my <arg2> have three <arg1>
5	$\texttt {N}\_\texttt {PURPOSE-OF}\_\texttt {N}\,{:}{:}{=} \texttt {com}<\texttt {arg2}>\texttt {sem} <\texttt {arg1}>$
	// with <arg2> without <arg1>
6	$\texttt {N}\_\texttt {PURPOSE-OF}\_\texttt {V}\,{:}{:}{=}<\texttt {arg1}>\texttt {para} <\texttt {arg2}>$
	// <arg1> for <arg2>
7	$\texttt {ADJ}\_\texttt {PROPERTY-OF}\_\texttt {N}\,{:}{:}{=} \texttt {a}<\texttt {arg2}>\acute{\texttt {e}} \texttt {branda e} <\texttt {arg1}>$
	// the <arg2> is soft and <arg1>

The strategy generates a poem according to a structure and a set of seed words. The latter restrict the semantic network only to relation instances involving them, with a probability set for going one level further. Textual lines are then produced from the selection of instances in the restricted network and the application of the grammar for rendering them. Such lines are scored by comparing their metre with the required for the lines, with a bonus for end rhymes. All syllable-related operations are done with the SilabasPT tool. The top-scored lines are used to fill each line in the poem structure.

Even though any semantic network with relation instances represented as triples would suit PoeTryMe, originally, it used CARTÃO (Gonçalo Oliveira et al., 2011), a network with about 332,000 relation instances, automatically extracted from three Portuguese dictionaries. CARTÃO was based on extraction grammars created with the PEN parser, the same parser used for the generation grammars of PoeTryMe, even if used in the opposite direction. Generation grammars can be extracted from any source of text, but collections of poems suit better the purpose. Moreover, as the relation arguments in CARTÃO are lemmas, in order to include also their inflection (e.g., pattern #1 in Table 1 requires arguments to be in the feminine, and #4 in the plural), the grammar creation process gets the gender and number inflections from LABEL-Lex (Ranchhod et al., 1999), a morphological lexicon for Portuguese.

**Fig. 4:**
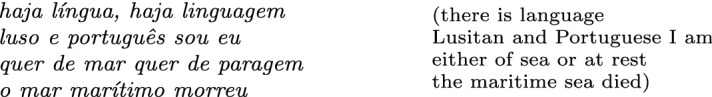
Block-of-four generated by PoeTryMe, using the seeds *mar* and *português*

Figure 4 has a block-of-four 10-syllable lines generated by PoeTryMe with the seeds *mar* and *português* (in English, ‘sea’ and ‘Portuguese’). In fact, the third line has only 7 metrical syllables, but it was selected due to the rhyme bonus. All the lines rhyme and use a pair of words, out of which one is a seed and the other is directly related (synonymOf(*português*, *luso*), partOf(*paragem*, *mar*), propertyOf(*marítimo*, *mar*)) except for the first line, where an indirectly-related word was selected (synonymOf(*língua*, *linguagem*), hypernymOf(*língua*, *português*)).

**Fig. 5:**
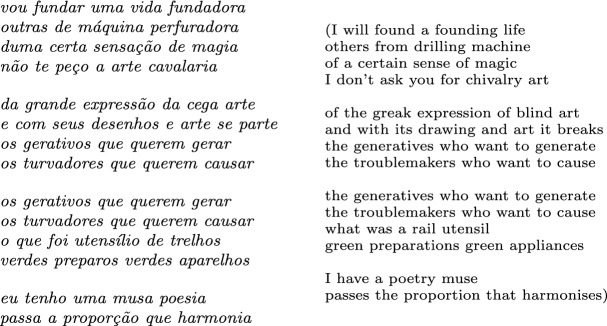
Poem generated by PoeTryMe in the form of a sonnet, using the seeds *poesia*, *arte*, *máquina*, *criar*, *gerar*

Figure 5 is a longer output, in the form of a sonnet, generated for the seeds *poesia*, *arte*, *máquina*, *criar*, *gerar* (‘poetry’, ‘art’, ‘machine’, ‘create’, ‘generate’). Again, all lines rhyme, but the fourth and the sixth have one more syllable than the target, which was 10.

Not that the poems have an extremely clear message, but, independently, most lines tend to follow a grammatical structure, acquired from human-written poetry, and to be semantically-coherent, because, even if different words are used, the semantic relation is preserved. Moreover, using the seeds or semantically-related words in the whole poem helps with cohesion. When compared to the semantics in Tra-la-Lyrics, this is a step forward.

### Instantiations of PoeTryMe

PoeTryMe has a flexible architecture, where the combination of lines according to a given form (generation strategy) is independent from the generation of lines, which is supported by the templates and the semantic network. This made possible its adoption in the development of different systems, targeting the generation of poetry with different underlying resources, though with different goals or interaction. For instance, PoeTryMe was the base for developing Tra-la-Lyrics 2.0 (Gonçalo Oliveira, 2015). The main difference was that, instead of a poem structure, the input was a melody that had to be analysed and then converted to a sequence of beats. There was also a higher focus on matching stressed syllables with strong beats.

Tra-la-Lyrics 2.0 was experimented with a feature of PoeTryMe not described in the previous section: the expansion of the given seeds with additional related words. This is achieved with the Personalized PageRank (Agirre & Soro, 2009) algorithm, based on random walks for ranking all the words in the semantic network according to a context (i.e., the seeds). The top-ranked words are then added to the set of seeds. More than expanding this set, this algorithm can be used for biasing the poem towards a sentiment, i.e., PoeTryMe may select only the top-ranked words that, according to a sentiment lexicon, have the desired polarity (positive or negative). SentiLex-PT, a polarity lexicon for Portuguese, is used for this purpose. This is illustrated for the seeds *amor* and *artificial*, in Table 2.

**Table 2:** Expanding the seed word *amor* with a bias on sentiment

Word	Bias	Expanded seeds
*amor*	None	*sentimentos*, *ódio*, *meda*, *cupido*, *humor*
		(feelings, hate, heap, cupid, humor)
	Positive	*sensualidade*, *amorosos*, *paixão*, *querido*, *afecto*
		(sensuality, loving, passion, dear, affect)
	Negative	*ódio*, *medo*, *cólera*, *terror*, *arrepio*
		(hate, fear, cholera, terror, shiver)
*artificial*	None	*artificialidade*, *carro*, *fingida*, *natural*
		(artificiality, car, fake, natural)
	Positive	*natural*, *sofisticada*, *concisa*, *esmerada*
		(natural, sophisticated, concise, emerald)
	Negative	*artificialidade*, *fingida*, *afectada*, *teatral*
		(artificiality, fake, affected, theatrical)

**Fig. 6:**
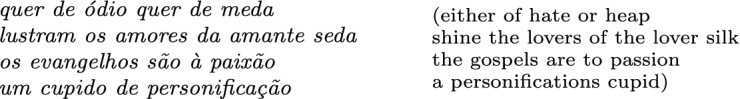
Lyrics generated by Tra-la-Lyrics 2.0 for the song *Alecrim*, with seeds expanded from *amor*

**Fig. 7:**
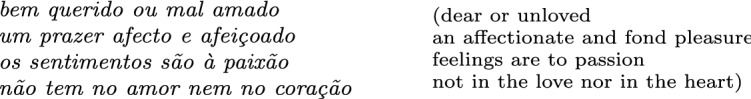
Lyrics generated by Tra-la-Lyrics 2.0 for the song *Alecrim*, with seeds expanded from *amor* with positive polarity

**Fig. 8:**
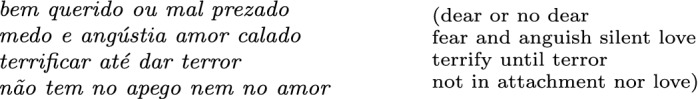
Lyrics generated by Tra-la-Lyrics 2.0 for the song *Alecrim*, with seeds expanded from *amor* with negative polarity

To illustrate both Tra-la-Lyrics 2.0 and the expansion feature, Figs. 6, 7 and 8 show three lyrics generated for the Portuguese popular song *Alecrim*, with the seed *amor*, respectively expanded with no polarity, positive polarity and with negative polarity.

Besides Tra-la-Lyrics 2.0, other instantiations of PoeTryMe include:

- Its adaptation to other languages, namely Spanish and English (Gonçalo Oliveira et al., 2017), which was mainly a matter of changing the following language-dependent resources: the semantic network was replaced by wordnets available for the target languages; the grammars were acquired from poetry written in the target languages; the morphology lexicon, the polarity lexicons and the syllable division tool were replaced by alternatives for the target languages.
- A bot on the Twitter social network, baptised as *O Poeta Artificial* (Gonçalo Oliveira, 2017a), which posts Portuguese poems inspired by recent trends.^3^ Briefly, words associated with the trends are used as seeds. Also, if they fit, templates mentioning the trend or lines by Twitter users can also be included in poems.
- An interactive web application, Co-PoeTryMe (Gonçalo Oliveira et al., 2019), which enables human users to interact with PoeTryMe in the collaborative composition of poetry.^4^

## Internet humor

Among all the humorous artefacts constantly spread through the Internet, so called Internet memes are certainly a popular form. Specifically, memes based on an image macro are a simple way of transmitting a funny idea by combining verbal and visual humor. So much that many templates emerged, meaning that there are several image macros and their textual templates suiting a broad range of situations. These can be used in different contexts, such as comments in social media or in advertising campaigns, often as an attempt to amplifying attention to the related content. Such memes are a product of human creativity and we see their automatic generation as a challenge for CC. After a brief overview of previous work on humour generation, this section presents Memegera, a creative system that produces Internet memes with Portuguese text, for given short texts, in this case, news headlines.

### Related work

Besides CC, automatic generation of verbal humour is also in the scope of Computational Humour (Amin & Burghardt, 2020). Notable work on the former includes the generation of: punning riddles based on rules and a lexicon with syntactic and semantic information (Binsted & Ritchie, 1997); funny acronyms, based on known acronyms, WordNet, rhyme and rhythm (Stock & Strapparava, 2006); adult humour, based on lexical replacement, considering taboo words, coherence, position and form (Valitutti et al., 2016); or one-liners, generated from human-rated examples (Winters et al., 2018).

Another task in the scope of Computational Humor for which there is some work in Portuguese is irony detection (Corrêa et al., 2021), humor recognition (Gonçalo Oliveira et al., 2020), and on the analysis of user comments to satirical news (Wick-Pedro et al., 2020). For Spanish, related work went further to rate and analyse humor (Chiruzzo et al., 2021).

Back to generation, to our knowledge, Memegera (Gonçalo Oliveira et al., 2016) was the first creative system developed with the goal of generating Internet memes. These artefacts combine verbal and visual humour, thus adding another dimension to humour generation. Since Memegera, research interest on these artefacts has been growing. By leveraging on deep neural networks and large collections of human-produced memes, with images, their class, and their original captions, humorous captions have been generated for image macros, e.g., with a CNN for image embedding and a (LSTM) RNN for language generation (Peirson V & Tolunay, 2018), and with a transformer-based encoder-decoder (Vyalla & Udandarao, 2020). Despite following a language-independent approach, the previous were tested in English, for which large datasets could be collected. Another difference towards Memegera is that the previous start with an image and do not select one for a given context, expressed in text. In addition to meme generation, there is also work on their automatic processing, including a shared task for sentiment analysis and emotion recognition in memes (Sharma et al., 2020).

### Memegera

Memegera (Gonçalo Oliveira et al., 2016) is a creative system that attempts to achieve a humorous effect by illustrating news headlines with automatically-created memes, based on a set of well-known image macros and using Portuguese text. Given a headline, Memegera: (i) selects a suitable image macro out of a set of covered macros; (ii) adapts the text according to the selected macro; (iii) adds the resulting text to the image macro. For an easier reference to image macros and their meaning, we suggest the Know Your Meme^5^ website, which documents Internet memes.

The selection of a suitable macro relies on a set of rule-based triggers, most of which based on the utilisation of a specific lemma or sequence of words in the headline, in some cases also considering the sentiment. Examples include:

- Announcements, denoted by lemmas of specific verbs (e.g., *anunciar*, *preparar*; in English ‘announce’, ‘prepare’), for the *Brace Yourselves* macro;
- Unfinished actions, denoted by *não* (‘no’) followed by a verb, for the *One Does Not Simply* macro;
- Opposing ideas, denoted by *ou* (‘or’), for the *Not Sure If* macro;
- Opinions, denoted by lemmas of specific verbs (e.g., *dizer*, *acreditar*, *achar*; ‘say’, ‘believe’, ‘find’) for *Condescending Wonka*;
- Negative phrase, followed by *mas* (‘but’) and a positive phrase, for *Success Kid*;
- Positive phrase, followed by *mas* (‘but’) and a negative phrase, for *Bad Luck Brian*.

Once a macro is selected, there are rules for obtaining the text to use, based on the headline text. Most of them fill a textual template with words or sequences from the headline. For the analysis of the headline and its transformation, different tools and resources are used. The headline is first analysed with the NLPPort (Rodrigues et al., 2018) pipeline, for tokenisation, PoS tagging and lemmatisation. Positive and negative phrases are identified with the help of SentiLex-PT (Silva et al., 2012), a polarity lexicon for Portuguese. In some cases, in order to fill the template, the words in the headline have to be inflected differently, with LABEL-Lex used for this purpose. In other cases, verbs need to be nominalised, with the help of the Nomlex-PT (de Paiva et al., 2014) lexicon.

**Fig. 9:**
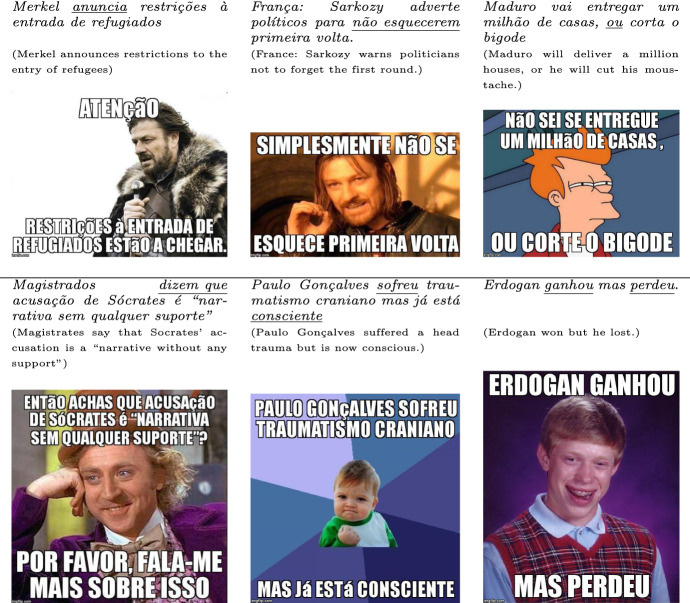
Examples of memes produced by Memegera

Figure 9 has a selection of headlines and memes following each of the aforementioned macros, all produced by Memegera. Triggers in the headline are underlined.

**Fig. 10:**
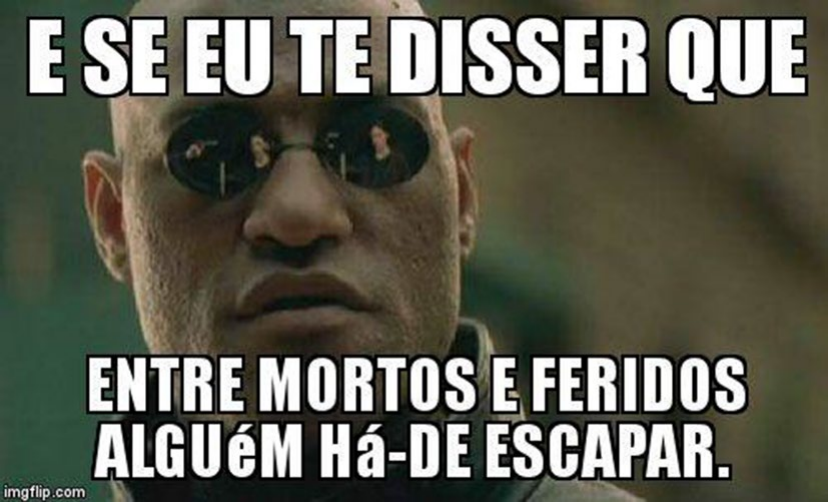
Example of the fallback meme *Matrix Morpheus* for the headline *Acidente faz nove feridos e condiciona trânsito no IC2* (Accident leaves nine wounded and limits traffic on IC2). Text translates to “What if I told you that among dead and wounded, someone will escape”

There are also fallback macros for headlines that do not trigger any other macro. One is *Matrix Morpheus*, starting with the text *E se eu te disser que...* (what if I told you) followed by a proverb with a high semantic similarity with the headline. Due to the lack of viable alternatives when Memegera was created, the computation of the semantic similarity between proverbs and headlines was based on the PMI-IR (Turney, 2001) measure, computed on the Portuguese Wikipedia. To restrict the number of candidate proverbs, only those using the most relevant word in the headline were considered. The most relevant word was approximated to be one that occurrs in CETEMPúblico (Rocha & Santos, 2000) newspaper corpus, but has the lowest frequency out of all content words in the headline. The tested proverbs were those available on the scope of the project Natura.^6^ Figure 10 has an example of this fallback meme for a specific headline.

For some time, until January 2018, Memegera was publishing regularly in its Twitter account.^7^ It came back some months later, posting only more simple memes based on Twitter trends, but it is not working anymore.

## Riddles

In opposition to memes, research on riddles is not so new. Riddles are verbal expressions, with one or more descriptive elements for which the referent is to be guessed (Georges & Dundes, 1963). More than general knowledge, the creation of riddles involves a creative exploitation of linguistic phenomena like polysemy, homophony and other kinds of lexical and semantic knowledge (De Palma, 1992). After a brief overview of related work, this section presents Seco, a creative system that generates riddles in Portuguese.

### Related work

Although not all riddles have a humorous intent, they can be adopted as a template for jokes, especially when formulated as question–answer, with the latter working as a punchline. An example are punning riddles (Binsted & Ritchie, 1997), produced by rules that exploited a lexicon with syntactic and semantic information, or others generated by mining incongruous circuits in a common-sense knowledge base (Labutov & Lipson, 2012). Besides the latter, riddles have been automatically generated from a knowledge base of famous characters and their properties (Guerrero et al., 2015), or by mixing word categories from a thesaurus and associated modifiers (Galvan et al., 2016). We should add that riddles produced by all the previous are in English.

### Seco

Seco (Gonçalo Oliveira & Rodrigues, 2018) is a creative system for generating riddles in Portuguese, following six different models. Since most results are not consensually humorous, they are framed as *piadas secas* (roughly, dry jokes), a term used in Portugal for jokes that are not that funny, even though, in some cases, the anti-climax may still result in laughter.

Riddle generation resorts to a lexico-semantic knowledge base similar to PoeTryMe’s semantic network (see Sect. 2.3), i.e., a graph of words connected according to different semantic relations between their senses. All generation models start with a given concept with two detachable parts, $c_1$ and $c_2$. This can be either a compound (e.g., “human rights”) or a single word that may be divided in two (e.g., “knowledge = know + ledge”). Relation instances where each part is involved are then retrieved from the knowledge base and exploited in the formulation of a question, following one of six models:

- Reinterpretation of compounds (RC): a literal interpretation of compounds (e.g., *primeira mão* or *ensino superior*; in English, ‘first hand’ or ‘higher education’), based on relations where its parts are involved.
- New compounds (NC): creation of new compounds with a similar sound as known compounds, i.e., with an edit distance of 1 (e.g., *amido oculto* for *amigo oculto*, or *primeiro pano* for *primeiro plano*).
- Reinterpretation of words (RW): similar to the reinterpretation of compounds but based on splitting single words (e.g., *centralidade = central + idade*, or *restolho = resto + olho*).
- New blends (NB): similar to the new compounds but for single words, after a small change in their orthography based on a handcrafted list of possible replacements (e.g., *bombesta = bom + besta* for *bombista*, or *fundasom = funda + som* for *fundação*).
- Partial antonyms (PA): splitting single words in such a way that, when alone, one of the parts has a known antonym, then used for the creation of a novel antonym (e.g., *pormaior* for *pormenor*, or *noitente* for *diante*).
- Antonymy blend (AB): similar to the reinterpretation of words, but focused on those for which both parts have a known antonym, leading to another process of creating a novel antonym (e.g., *odiar-atingir* for *amarfalhar*, or *mau-mau* for *bombom*).

To finally produce a riddle, it is necessary to combine the generated and the original concepts with related concepts in a question-answer format. For this, there is a small set of templates for describing each relation in natural language and rules for putting everything together. Seco’s implementation was supported by a knowledge base that includes relations from ten Portuguese lexical resources (Gonçalo Oliveira, 2018). For higher confidence and familiarity with the relations, this was restricted to a subset of about 45,000 relation instances found in three or more resources. However, as the relation arguments in the previous knowledge base are in the lemma form, which would limit the number of possible combinations and interpretations of words, LABEL-Lex was used to consider inflections. Moreover, for testing the first and the second model, inputs were obtained from two sources: a list of 180 Portuguese noun-adjective compounds (Ramisch et al., 2016); and 289 noun-adjective pairs with at least 750 occurrences in the CETEMPúblico (Rocha & Santos, 2000) newspaper corpus.

Table 3 illustrates the outputs of Seco with several riddles and the models they instantiate. There are interesting results, which explore novel associations. Some have humour potential, but others would require further polishing.

**Table 3:** Examples of riddles generated by Seco and model followed

Riddle	Model
*Que resulta do cruzamento entre um plano e um homem? direitos humanos.*	RC
*Qual é o contrário de pagamento seco? sinal verde.*	RC
*O que significa primeiro sinistro? O que é inicial e mau.*	NC
*O que significa porto forte? um vinho que é violento.*	NC
*Que resulta do cruzamento entre o que é calado e o que é comum? mudo geral.*	NC
*Que resulta do cruzamento entre a finalidade de lixa e um treinador? politécnico.*	RW
*O que significa solícito? uma estrela que não é/está ilegal.*	RW
*O que significa cãotributo? um pagamento canino.*	NB
*O que significa ecoponto? um som que serve para coser.*	NB
*Qual o contrário de bombeiro? maubeiro.*	PA
*Qual é o contrário de prevenir? prevandar.*	PA
*Qual é o contrário de atropelado? alvo-vestido.*	AB
*Qual é o contrário de pobre velho? novo-rico.*	AB
*Qual é o contrário de virtual? irtual.*	AB
*Qual é o contrário e animal? anibem.*	AB

## Headlines

More than a mere title of a story, headlines are an invitation to reading its content. So, with the amount of information currently spread on the Web, it is crucial to have appealing headlines, which should contribute to attract more readers. This would apply to any kind of story, from news to advertising, and the effect would be similar to illustration with an Internet meme like those of Sect. 3. The main difference is that memes will have the visual component. Yet, without this component, the form of the produced text is less restricted and the result may thus be more surprising. Since the production of appealing headlines requires both time and creativity, it is a suitable task for CC. After a brief overview of related work, this section presents TECo, a system for adapting known expressions to original headlines.

### Related work

For higher memorability, the automatic generation of headlines often resorts to well-known expressions, with which readers are familiar, such as slogans, movie or song titles. This can be done by replacing words in the expression with keywords from an original human-written headline (Gatti et al., 2015), or by injecting such expressions in headlines by an automated journalism system (Alnajjar et al., 2019). Previously generated metaphors may also be paired with the current news (Veale et al., 2017).

The generation of creative headlines is closely related to the generation of slogans, which also have to be short and appealing. Here, slogan templates have been filled with terms acquired from documents of two distinct domains (Repar et al., 2018), or words grammatically-related to a target concept and a generated metaphor for a property (Alnajjar & Toivonen, 2020). In any case, stylistic devices like rhyme and alliteration are also considered.

Recently, neural architectures have also been adopted to the automatic generation of funny and satirical headlines. Some related work was driven by the release of Humicroedit (Hossain et al., 2019), a dataset of original headlines, their human-edited versions to make them funny, and their rate according to other humans. An example (Winters & Delobelle, 2021) evolved headlines with the help of two transformer models (RoBERTa) fine-tuned in the previous dataset: a masked language model, trained on the text sequences, used in the mutation operator; and a regression model, learned from the humor ratings, used as fitness function. However, despite being minimal, the edits in Humicroedit may change the meaning of the original headlines. In a different work (Horvitz et al., 2020), a model for abstractive summarisation is learned from satirical headlines and their context, which includes both the content of the story and Wikipedia articles on mentioned entities. To some extent, this adds the humor aspect to the related task of automatic headline generation for a given textual document (Takase et al., 2016).

### TECo

TECo (Mendes & Gonçalo Oliveira, 2020), standing for *Texto Em Contexto*, is a creative system for adapting known expressions (e.g., proverbs or movie titles) according to an original headline. The result may be used as a more creative headline, as a sub-title, or just as a comment in social media. This is also a commonly adopted strategy in news satires, such as the *Daily Show*, in the US, or *Isto é Gozar com Quem Trabalha*, in Portugal. Examples in the latter show include the expression “Droga de Elite” (‘Elite Drug’, 5th April 2020), an adaptation of the Brazilian movie title *Tropa de Elite* (‘Elite Squad’), for a story on Covid-19 in the Brazilian favelas, where drug dealers were ensuring that residents followed the sanitary rules; or “Luzes, Câmara, Estupefação!” (‘Lights, Camera, Awe!’, 21st July 2020), an adaptation of the known expression *Luzes, Câmara, Ação!* (‘Lights, Camera, Action!’), when the Portuguese President admitted he was speechless when he found out that the money to inject in a bank was more than originally expected.

In order to adapt expressions (from a list) to an input text (e.g., headlines), three different methods were implemented in TECo, namely:

- Substitution (Sub) replaces the most relevant word in an expression, *a*, by a word from the input, *b*, or a similar one. This method can be applied to most expressions, and may thus be used as a fallback for the other methods, i.e., when no other adaptation can be performed.
- Analogy (An) relies on a common operation for computing analogies in word embeddings ($b - a + a^{\prime} = b^{\prime}$) (Mikolov et al., 2013), phrased as $b'$ *is to* *b* *as* $a'$ *is to* *a*. This method replaces the two most relevant words of the expression, *a* and $a^{\prime}$, with the most relevant word in the input, *b*, and another that is related to *b*, $b^{\prime}$, analogously as $a^{\prime}$ is to *a*.
- Vector Difference (VD) also selects the most relevant words in the input, *b* and $b^{\prime}$, but then: (i) computes the vector between them, $b - b^{\prime}$; (ii) identifies the pair of content words in the expression, *a* and $a^{\prime}$, that maximise $cosine(b-b^{\prime}, a-a^{\prime})$; (iii) replaces *a* and $a^{\prime}$, respectively by *b* and $b^{\prime}$.

Table 4 illustrates each method with examples of headlines, proverbs, and the resulting expressions. Replaced words and their replacements are underlined. More precisely: in the first example, produced with the Substitution method, $b= bancos$ replaces $a= amigos$; in the second, by Analogy, $comeces= apontar - deixes + fazer$; in the third and fourth, both by VD, $fere - ferido \approx finge - detido$ and $m\tilde{a}o - feliz \approx m\acute{a}scara - est\acute{u}pido$.

**Table 4:** Running examples of the application of each adaptation method of TECo

	Headline	Proverb	Output
Sub	*Bancos* *preparam-se para dar menos crédito às famílias* (Banks preparing to give less credit to families)	*amigos*, *amigos*, *negócios à parte* (Friends, friends, business apart)	*bancos*, *bancos*, *negócios à parte*(Banks, banks, business apart)
An	*EUA estão a* *apontar* *para o pior número de desemprego da sua história* (USA are pointing out to the worst unemployment numbers in their history)	*não* *deixes* *para amanhã o que podes* *fazer* *hoje* (Do not leave for tomorrow what can be done today)	*não* *comeces* *para amanhã o que podes* *apontar* *hoje* (Do not start tomorrow what you can point out today)
VD	*Finge* *ter Covid-19 no Facebook e acaba* *detido* (Pretends to have Covid-19 on Facebook and ends up arrested)	*quem com ferro* *fere*, *com ferro será* *ferido* (Those who hurt with iron, with iron shall be hurt)	*quem com ferro* *finge*, *com ferro será* *detido* (Those that pretend with iron, with iron shall be arrested)
VD	*“Um erro* *estúpido*”. *Primeira-ministra escocesa pede desculpa por não usar* *máscara* (Pretends to have Covid-19 on Facebook and ends up arrested)	*não está sempre na* *mão* *de cada um o ser* *feliz*, *mas está o merecê-lo.* (Those who hurt with iron, with iron shall be hurt)	*não está sempre na* *máscara* *de cada um o ser* *estúpido*, *mas está o merecê-lo.* (Those that pretend with iron, with iron shall be arrested)

Additional examples by TECo can be read in its account in the Twitter social network^8^ where, from time to time, TECo reads headlines in the Portuguese press and retweets one, together with an adapted expression. It initially tries to use the VD method but, if it cannot find a suitable pair of words for replacing, also considering their PoS, it tries the other two methods. The running implementation has a knowledge base with about 4600 known expressions, out of which 1,600 are Portuguese proverbs, collected in scope of the project Natura,^9^ and 3000 are movie titles in Portuguese, collected from the IMDB dataset.^10^ Using expressions from the folk and pop culture is a step towards making the headline familiar and thus more appealing.

Out of all the available expressions, only one is posted for each headline. So, TECo has mechanisms for selecting the expressions to adapt and, when more than one result is produced, the one to post. Before adaptation, it may rank all the expressions in the knowledge base according to their semantic similarity with the headline. In this process, several methods can be used for representing the text, from Jaccard Similarity or TF-IDF vectors, to sentence embeddings based on GloVe (Pennington et al., 2014) or BERT (Devlin et al., 2019). Adaptation is then only tested in a subset of 300 expressions that includes the most similar ones and others randomly selected, for higher diversity. This step alone may also be used to simply suggest the expressions for the headlines, without further adaptations, so we can see it as an alternative to the fallback mechanism of Memegera (see Sect. 3). The same methods are used for selecting the top expression out of all that result from the adaptation process. Only this one is posted.

TECo also uses a model of GloVe word embeddings (Pennington et al., 2014) pretrained for Portuguese (Hartmann et al., 2017). This model is used in the representation of words as vectors, thus enabling the computation of similar and analogously-related words, but also to identify the most relevant words in both headlines and expressions. Although different methods could be used for this, namely TF-IDF, we took advantage of the fact that the words in the GloVe vocabulary are ordered according to their frequency. Having in mind that relevance tends to be indirectly proportional to document frequency, we consider the lowest-ranked content words to be the most relevant. The LABEL-Lex morphology lexicon is used for identifying content words and for providing information on word inflection, thus enabling that replacements have the same inflection as the words they replace.

## Discussion

The generation of different types of text has different requirements at different levels of language, from sound and form, to syntax and semantics. For producing text in Portuguese following such requirements, the overviewed systems had, to some extent, to be tailored for the available linguistic resources for this language.

In poetry and song lyrics, form and metre play an important role, together with rhymes. Metre is tightly connected to the number of syllables and stress position, which is handled by a rule-based tool, SilabasPT, also used for rhyme identification. In a language like Portuguese, this is straightforward because, despite a minority of exceptions, the same sequences of characters will have the same sound. This is similar to other languages, like Spanish, but not for English, where a pronunciation dictionary would be needed (Gonçalo Oliveira et al., 2017).

Depending on the starting point, there are different levels of freedom for generation. In the first version of Tra-la-Lyrics, selecting one word at the time provided great control on the local metre, but not so much on the overall output, in terms of syntax and, especially, semantics. On the other hand, selecting and adapting complete lines considerably narrows the space of possible results, and thus surprise, but provides higher control on the overall form and message, also resulting in higher cohesion. Decisions regarding the trade-off between freedom and control of some variables are common in the development of creative systems. For instance, adapting a full poem, by only replacing its content words, will limit the possible generations as a trade-off for higher cohesion. Rather than generation, this strategy is often referred to as text adaptation or transformation.

Despite their differences, both riddles and meme text follow pre-defined templates, which already meet formal constraints. Yet, even when created by humans, the previous already tend to be constrained by a set of common forms. For headlines, formal constraints are more relaxed. They only have to be short enough, which is guaranteed once they result from short expressions.

In fact, as it might happen for human creativity, most creative systems do no create completely from scratch. They are inspired by existing artefacts, often learning from them, reusing or re-combining parts of them, or simply aiming at their adaptation to a new context.

Templates are also useful for controlling syntax. As long as they are syntactically accurate, we are more than halfway towards producing syntactically-correct outputs. But this is only fully achieved if filling words suit the correct PoS and inflection. For this, morphology lexicons are extremely useful. Specifically for Portuguese, the development of the overviewed systems would not be possible without a resource like LABEL-Lex, which can be used for obtaining the lemmas of the words and their possible PoS, as well as their gender and number inflections, or verb conjugations.

Syntax will have a positive impact on semantics, but it is not enough, as we have shown, for instance, in the first version of Tra-la-Lyrics. For increased semantic coherence, poetry generation relied on a semantic network, where words are connected according to their possible senses (Gonçalo Oliveira, 2018; Gonçalo Oliveira et al., 2011). For riddles, such a resource is key, because it is where words and their features are acquired from.

Yet, a semantically-coherent text is not exactly the same as one with a coherent message or one suitable for a given input context. Poetry has been generated for a set of seed words, which somehow define a topic, also used to constrain the semantic network. While this indeed guides generation through the desired topic, it is not enough for transmitting a clear message. However, poetry is often rich in figurative language, and typically has an open interpretation. So, even if there is room for improvement, we argue that this mix of semantically-coherent lines on the desired topic and a regular metre, possibly including additional poetic features in the line templates, gives a good enough sense of poetry (i.e., a text that most people would associate to a poem).

For both memes and headlines, input context is given as a headline in a newspaper, which is more specific than a set of words. Most triggers for the memes are simply based on the utilisation of certain patterns, so semantics does not matter much in the end. Yet, a minority is based on the sentiment, analysed with the help of a sentiment lexicon. As it happens for the generation of poetry or lyrics, SentiLex-PT is the elected source of information on the polarity of Portuguese words. Where semantics is more important is probably in the fallback meme, which selects a suitable proverb for a headline, based on their semantic similarity. Specifically in Memegera, semantic similarity is computed on-the-fly, with PMI-IR (Turney, 2001) on Wikipedia. A suitable alternative would resort to a vector representation of sentences and on the cosine similarity. As it happens in TECo, this could be as simple as TF-IDF vectors (e.g., based on the collection of proverbs), or resort to static word embeddings (e.g., word2vec or GloVe), or sentence embeddings from BERT. In addition to computing similarity with the input context, TECo further exploits static word embeddings for replacing words in an expression with others from the context that are analogously-related to the replaced words. This does not only increase relatedness with the context, but contributes both to semantic and syntactic coherence. For Portuguese, we have used word embeddings from the NILC repository (Hartmann et al., 2017).

Top similar words computed from static word embeddings would also be a suitable and faster alternative to PoeTryMe’s seed expansion procedure. In theory, they could even replace the semantic network in both PoeTryMe and Seco, with relations computed as analogies, like in TECo. Currently, Seco generates a bulk of riddles for the given resources. Not being limited by explicit relations in the semantic network could open the door to riddle generation for a given context, e.g., based on an extensive search of related words.

What also helps to increase relatedness with the context is the ability to discriminate between relevant and irrelevant words. Content words are often easy to identify with a morphology lexicon or a PoS tagger. To select the most relevant, methods like TF-IDF are necessary. However, to be useful for this purpose, the corpus for computing TF-IDF must be large enough. An alternative is to use an external corpus. When we do that, the frequency of a word in the corpus is already a good indicator of its relevance (i.e., the lower the frequency, the higher the relevance), and easier to get than the whole corpus. This is why, for computing word relevance, Memegera relies on frequency lists, in this case from CETEMPúblico (Rocha & Santos, 2000), and TECo on the vocabulary of the word embeddings, which is ranked according to frequency in the large corpus they were learned from.

All the previous resources provide the necessary linguistic knowledge and contribute to the development of systems that go beyond the simple generation of random text. Whether in the form of a list of words or of a complete sentence, the input has a noticeable impact on the generated text, confirming that these systems can produce a large number of artefacts, applicable to different situations. More than that, they have the ability to rank their productions (e.g., poems according to the metre and rhyme; riddles according to corpus frequency and number of related words), sometimes also considering the given context (e.g., headlines). This is further tested when these systems operate as a Twitter bot and continuously exhibit their skills by generating and posting artefacts inspired by up-to-date topics.

Still on the underlying resources, they are key for generating text in the target language, in this case, Portuguese. So much that the systems were tailored for these resources. To some extent, we believe that they could be adapted to other languages, especially Latin languages, as long as similar resources and tools exist for such languages. We have mentioned the adaptation of PoeTryMe to Spanish and English, detailed elsewhere (Gonçalo Oliveira et al., 2017), but this would still apply to Memegera, Seco, and TECo, if the corpora, lexicons, semantic networks and distributional models are replaced by some on the target language. For Memegera and Seco, some work would also be required for rewriting the rules at the surface level of text and translating the used templates to the target language; and for Memegera, a PoS tagger would be necessary. Still, we suppose that, if creative systems were developed from scratch for other languages, the available resources would be considered from early stages of development, which could result in significantly different systems. It is also important to stress that, more options would be available for higher-resourced languages, like English.

A final word should be given on the evaluation of the overviewed systems. In opposition to other tasks, the quality of creative language is highly subjective, and the evaluation of creativity is highly dependent on human perceptions (Jordanous, 2017). So, evaluation is hardly automatised. For PoeTryMe, less subjective aspects of poems were assessed (Gonçalo Oliveira et al., 2017). It was concluded that, as intended, PoeTryMe can generate poems that: are semantically-associated with the given seeds; have a regular metre and frequent rhymes; and have a good degree of variation. However, these conclusions are limited to the test setting, whereas, by simply replacing the underlying linguistic resources (mainly the grammar and the semantic network), PoeTryMe could easily become a different system.

Yet, despite known limitations, evaluation is typically based on human opinions, which also makes comparison with the state-of-the-art difficult. Depending on the creative artefact, humans can be asked different questions. For the overviewed systems, we highlight the following human evaluations and their main conclusions. Lyrics by Tra-la-Lyrics 2.0 were considered to be better than those by the original system, in terms of all human-assessed aspects, namely rhythm, rhymes, sound, grammar, meaning, topic and overall appreciation (Gonçalo Oliveira, 2015). Memegera productions are as surprising as Internet memes by humans, but, whereas still rated positively, not as coherent or funny (Gonçalo Oliveira et al., 2016). Riddles by Seco are, to some extent, interpretable, surprising and, especially, novel, while having some humor potential (Gonçalo Oliveira & Rodrigues, 2018). Headlines by TECo (Mendes & Gonçalo Oliveira, 2020) are generally syntactically correct; about 60% are somewhat or clearly related to the input; despite reusing known expressions, close to 50% were classified as completely novel, especially with the VecDiff method; and about 20% were very funny.

## Embracing new trends

All overviewed systems for generating creative text in Portuguese rely on some kind of rules and on the exploration of linguistic resources to achieve their goal. So, we can say that text generation approaches based on deep neural networks are under-explored for this language. In this section, we discuss how such approaches could be adopted for Portuguese, and include some recent experiments with more recent models.

We should start by noting that most neural models are language independent. So, except for a corpus of the target kind (e.g., poetry, riddles) in the target language, they typically do not require any external linguistic resources. The corpus is used for training the model, which will learn to generate text in a similar style. Nevertheless, in some cases, collecting and organising this corpus can be challenging, due to the lack of human-produced examples.

After RNN-based language models, transformers became the trend, with models like GPT2 (Radford et al., 2019) and GPT3 (Brown et al., 2020) being known for their text generation capabilities. They are often pretrained with general text and can be fine-tuned with text in a more specific style. However, even if generated text is fluent, with the standard models, there is not much control on features like the topic of the poem, sentiment or position of rhymes. It is only possible to provide a starting sequence (prompt), which can be the title, the first line of the poem or, as we will show ahead, a description of the task to perform.

We have explored the previous approach with GPT2-small, which has 124 M parameters and was pretrained in English text. The experiment consisted of collecting a 20 MB corpus of Portuguese poetry from Wikisource,^11^ and using it to fine-tune the previous model.

**Fig. 11:**
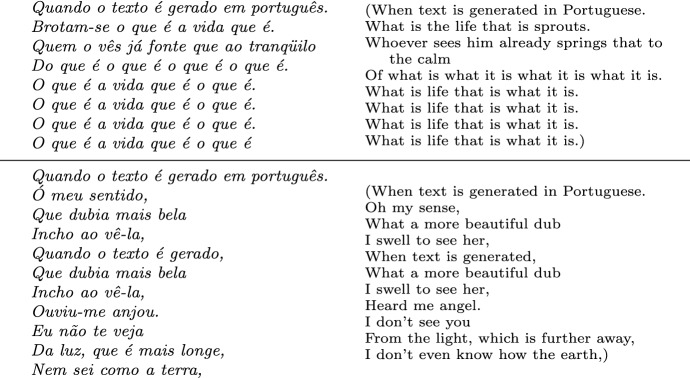
Examples of text produced by GPT2 fine-tuned in Portuguese poetry, with prompt *Quando o texto é gerado em português*

Figure 11 has two examples of text generated by the fine-tuned model, given the prompt *Quando o texto é gerado em português* (in English, ‘when text is generated in Portuguese’), with temperature 0.7, a recommended value for this parameter. Even if examples suggest a form of a poem, rhymes are scarce and metre is not that regular. Moreover, there are odd lines and a number of generations ends up repeating the same line several times, as in the first example. Deciding when to stop generation can also be tricky. Of course, these examples might have suffered from fine-tuning a model pretrained for English. Also, due to the specificities of poetry, we suspect that the model would benefit from a larger training corpus, possibly restricted to one or a small set of poem forms, as in Liao et al. (2019). This would be the case of many neural approaches, even if, in the case of transformers, pretraining with other kinds of text in the target language could minimise the amount of data necessary for fine-tuning.

A much more powerful model is GPT3 (Brown et al., 2020), which was pretrained in a larger quantity of text, covering different languages, and has a version with 175B parameters. Although GPT3 can be fine-tuned, it is better known for its application in scenarios of zero and few-shot learning, current trends in machine learning. Towards these, the model performs a task described in a textual prompt, given no specific training data (zero-shot), or only a small set of examples (few-shot). GPT3 is only available through OpenAI’s API,^12^ and its usage has an associated cost, but we could use free credits for experimentation in the previous scenarios.

**Fig. 12:**
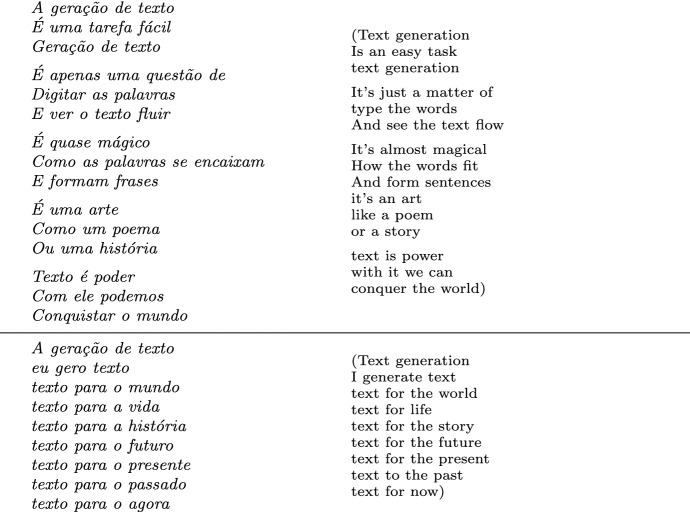
Examples of text produced by GPT3 with the prompt *Escreve um poema sobre geração de texto*

**Fig. 13:**
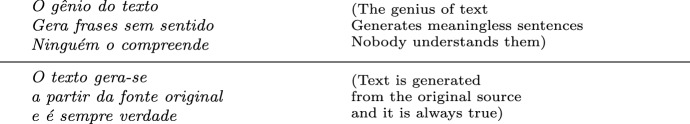
Examples of text produced by GPT3 with the prompt *Escreve um haiku sobre geração de texto*

The first experiment was again on poetry generation, in a zero-shot scenario. Using a prompt exactly like the one used with GPT2 would not be enough in this scenario. However, if the prompt specifies the task to perform, interesting results can be obtained. Figure 12 illustrates this for the prompt ‘*Escreve um poema sobre geração de texto.*’ (in English, ‘Write a poem about text generation.’), with temperature 0.7. Since the prompt may include the generation intent, we can say that there is some control on the generated content. Content is fluent and related to the topic included in the prompt (text generation). As it happens to GPT2, metre is not that regular, even if text seems to approximate possible forms of poetry. Other experiments were performed with a similar prompt but replacing the word *poema* (poem) by a form of poetry (e.g., *quadra*, *soneto*) but GPT3 would often fail on the right number of lines, and rarely match the target metre. This is still interesting for a zero-shot scenario. Unless metre and rhymes are specifically modelled (e.g., as in Zugarini et al., 2019), text generation models will struggle to match specific forms of poetry. An exception happened for the shorter form of haiku, which always resulted in a three-line text and showed a trend to match the 5–7–5 syllable pattern. Two examples are displayed in Fig. 13.

As mentioned in Sect. 6, metre is better controlled if, instead of generated from scratch, text is transformed by replacing some words towards a goal. Here, masked language models can be used for suggesting replacement candidates. Indeed, in order to increase the number of rhymes, they have been used for suggesting replacements for the last words of each line in song lyrics (Nikolov et al., 2020), and for mutating headlines (Winters & Delobelle, 2021).

In this context, we have explored the masked language model of BERT (Devlin et al., 2019) for transforming human-written lyrics (Gonçalo Oliveira, 2021). Briefly, the content words in an original poem can be masked and BERT used for predicting suitable words for the masks. Since BERT can suggest a ranked list of replacements, these can be filtered according to the end goals, i.e., grammatical and metrical constraints. BERT embeddings can also be used for computing similarity between produced lines and a topic, as long as described as a sequence of words.

**Fig. 14:**
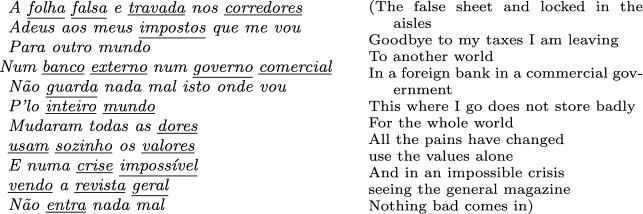
Lyrics of the song *Contentores* transformed with BERTimbau for the topic *criatividade computacional*

**Fig. 15:**
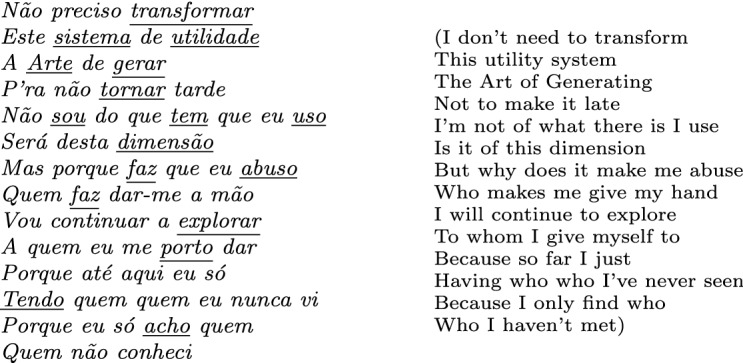
Lyrics of the song *Estou Além* transformed with BERTimbau for the topic *portal das finanças*

Figures 14 and 15 are examples of the previous approach using BERTimbau (base) (Souza et al., 2020), a BERT model pretrained for Portuguese, with 12 layers and 110 M parameters. Only content words can be replaced, and they are underlined in the figures. All functional words are kept. The first example is a transformation of the lyrics of *Contentores*, a song by the Portuguese band Xutos & Pontapés, with the topic *portal das finanças*, the name of the website where Portuguese contributors declare their taxes. The second results from the same procedure in the song *Estou Além*, by Portuguese singer-songwriter António Variações, with the topic *criatividade computacional* (computational creativity). The variable proportion of replaced words depends on how easy it is to find words related to the topic that match the metre of the original word. For the last words of each line, there is also a rhyme constraint, which is why the rhyme pattern of both examples is kept. Following the same metre and rhyme pattern makes it possible to sing the same songs with these new lyrics, which can be used as parodies, for mere entertainment or in advertising.

As it happens for poetry, to use neural approaches in the generation of other types of creative text, an important step would be the collection of enough training data, which can be challenging for some types of text. Here, riddles would be easier to collect than memes and their captions, because there are several dedicated websites and a dataset for humor recognition in Portuguese (Gonçalo Oliveira et al., 2020), where they could be picked from. However, if we think of specific types of riddle (e.g., “what do you get when you cross X and Y”? or “what is the opposite of X?”), only small amounts of human produced riddles can be found for each type. An alternative would be to generate riddles of the target kind with a tool like Seco and use them as training data, or instead to try few-shot learning.

Following this, we also experimented with GPT-3 for riddle generation. We first tried zero-shot, with the prompt *Conta-me uma piada sobre geração de texto.* (‘Tell me a joke about text generation.’). Results were variable and included some jokes in English, but also short stories in Portuguese, with some humor potential.

**Fig. 16:**
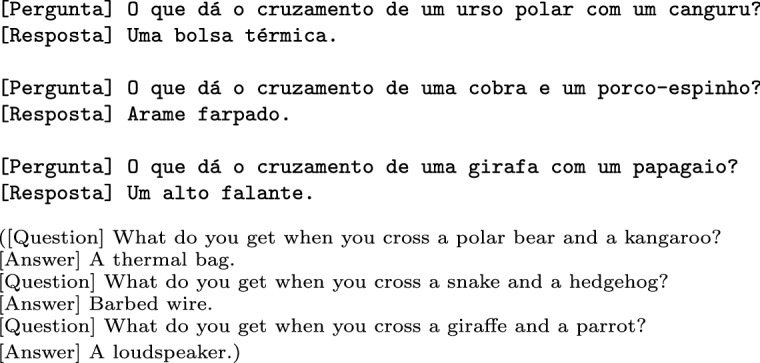
Prompt used for riddle generation in GPT-3

**Fig. 17:**
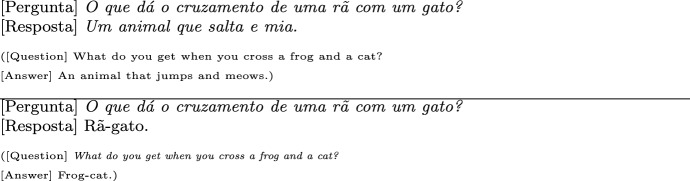
Selection of riddles generated when using three human-produced riddles as prompt

However, to start generating text that looked like riddles, we selected three human-produced jokes and put them together in a prompt to be used in few-shot learning (see Fig. 16). With this prompt and nothing else, a new riddle, like those in Fig. 17, was generated from scratch. They have a similar form, and the model could infer that all riddles have the name of two animals, changed for each question. In our exploration, for some reason, it insisted in using ‘frog’ as one. The answers made sense, but were too literal to be funny.

**Fig. 18:**
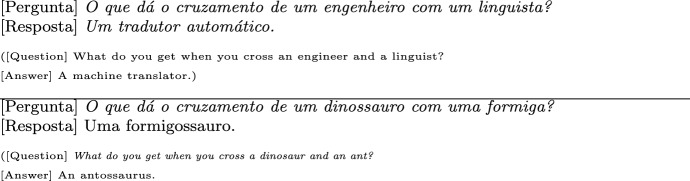
Selection of riddles generated when using three human-produced riddles as prompt

The model was also run with the previous prompt and a new question appended, such as those in Fig. 18. In this case, only the answers, also in the figure, were generated. While we cannot say that riddles generated this way are clearly funny, this approach virtually works for any riddle of this type. Moreover, some generated answers, such as those presented, are positively surprising, while still making sense. So, we confirm that GPT3 can generate text in the style of a small set of examples. However, as it happened to metre and rhyme in poetry, features like humor would benefit from specific modelling or ranking, e.g., by a humor classifier learned from human-rated examples (Winters & Delobelle, 2021).

When it comes to headlines, a similar few-shot approach could be adopted. It would only require a small set of original headlines paired with their creative version. As it happens for the riddles, a small number would be enough, and they could be created manually. An alternative would be to use results by TECo, which could also provide training examples for a more traditional language-modelling approach. Generated headlines could be finally ranked with a humor classifier which, for Portuguese, could rely on the available dataset (Gonçalo Oliveira et al., 2020), because half of its positive examples are humorous headlines.

This sums up the experiments with neural approaches for creative text generation in Portuguese. They were mainly shallow explorations and strong conclusions cannot be taken, but they open the door to a future generation of these systems.

## Conclusion

Computational systems for generating four distinct types of creative text, always in Portuguese, were overviewed. Tra-la-Lyrics is an earlier system for generating song lyrics, and PoeTryMe is a platform for generating poetry with semantic concerns. The latter has a flexible architecture that enabled its adaptation to different purposes. Memegera creates Internet humour as a combination of suitable image macros and text generated for a given headline. Seco generates riddles from a lexical knowledge base and a pair of concepts. Finally, TECo adapts known expressions, like proverbs or movie titles, to a context provided as a short text. Developing these systems would not be possible without a set of linguistic tools and resources available for Portuguese, which we highlight and discuss after describing all systems.

Besides suggesting alternative usages of computational linguistic resources, we have surveyed the fascinating area of linguistic CC, with a focus on Portuguese. This should contribute to disseminate the area and, hopefully, to a future increase of computational systems and approaches that generate creative text in Portuguese.

The truth is that there is still much to explore. State-of-the-art text generation models, based on deep neural networks, are under-explored when it comes to creative artefacts in Portuguese. Following this, we explored some models for text transformation and in the scenario of zero and few-shot learning, in what can be the starting point of a new generation of such systems. Moreover, there are other tasks in the domain of linguistic CC which are yet to be tackled for Portuguese, such as the generation of metaphors (Veale & Hao, 2007), neologisms (Smith et al., 2014), or narratives (Gervás et al., 2005).

We also note that a relevant difference between neural models and rule-based approaches is that the former are black-box, whereas the decisions of the latter are interpretable. This means that, despite being more limited in terms of outputs, rule-based approaches are easier to scrutinise and debug. Specifically, PoeTryMe goes beyond that and has a contextualizer module (Gonçalo Oliveira et al., 2017) for explaining the underlying decisions. This is achieved by providing the relations used for generating each line and their connection with the seed words. Therefore, an interesting avenue for future work in CC would involve combining the best of both approaches.

More than combining random words and fragments of text, the overviewed systems have shown that they can adapt to different situations. This means that they can be used in the autonomous creation of something new, specifically for some event, such as a celebration; and they can result in a funnier way of following recent news, possibly amplifying their range. The latter also applies for marketing and advertising in general, especially if we look at the application of CC to slogan generation (Alnajjar & Toivonen, 2020).

But the applications of CC are not limited to the previous. If AI aims at developing computational systems that exhibit human-like cognitive skills, creativity has to be one of its goals. So, embedding creativity in current intelligent systems will make them more human. For instance, a creative chatbot may improve user engagement by creating new jokes for a specific context, on-the-fly. Even for task-oriented chatbots, creativity might be an alternative way of dealing with out-of-domain interactions.

In most cases, engagement is more effective if the system is not completely autonomous and the human has an active role in the creation process. The area of human-computer co-creativity (Kantosalo et al., 2014; Liapis et al., 2016) is precisely about the collaboration of humans and machines in the creation of novel artefacts, ideally taking the best out of each intervenient in the process. In fact, a co-creative system has been developed in top of PoeTryMe (Gonçalo Oliveira et al., 2019). Once dedicated to creative writing, such a system may help creators deal with the ‘blank page syndrome’, e.g., simply by generating some initial lines, possibly guided by some topic, thus saving some time. Such systems may also have educational purposes, e.g., by making poetry composition more accessible to children (Kantosalo et al., 2015) or others otherwise not interested; or help fostering creative thinking (Liapis et al., 2016); not to mention therapeutic benefits of co-creativity in general (Bodily, 2020). At the same time, when it comes to evaluation, this scenario poses additional challenges (Jordanous, 2017) to the already challenging evaluation of creative systems.

We could certainly think of co-creative variations of the other overviewed systems. For instance, humans could collaborate with TECo in the production of more creative headlines. The human would have the role of making the initial selection of expressions and the final decision. TECo could be responsible for the adaptation, possibly going through different iterations where the human would rate the generated examples. This would also guarantee that every content would be reviewed by the human before publication, minimising the risk of transmitting an undesired message. So, besides adopting new trends and tackling other linguistic CC tasks, the development of co-creative systems should definitely be seen as another future research direction.

## Footnotes

1 https://abcnotation.com/.

2 https://code.google.com/archive/p/silabaspt.

3 https://twitter.com/poetartificial.

4 https://poetryme.dei.uc.pt/~copoetryme/.

5 https://knowyourmeme.com/.

6 https://natura.di.uminho.pt/~jj/pln/proverbio.dic.

7 https://twitter.com/Memegera.

8 https://twitter.com/TextoEmContexto.

9 https://natura.di.uminho.pt/~jj/pln/proverbio.dic.

10 https://www.imdb.com/interfaces/.

11 https://wikisource.org/.

12 https://openai.com/.
